# Effects of Zinc Oxide and Arginine on the Intestinal Microbiota and Immune Status of Weaned Pigs Subjected to High Ambient Temperature

**DOI:** 10.3390/ani10091537

**Published:** 2020-08-31

**Authors:** Se Young Yoon, Soo Jin Sa, Eun Seok Cho, Han Seo Ko, Jung Woo Choi, Jin Soo Kim

**Affiliations:** 1 College of Animal Life Sciences, Kangwon National University, Chuncheon 24341, Korea; ysy3532@naver.com (S.Y.Y.); Kofeed@kangwon.ac.kr (H.S.K.); jungwoo.kor@gmail.com (J.W.C.); 2 National Institute of Animal Science, Rural Development Administration, Cheonan 31000, Korea; soojinsa@korea.kr (S.J.S.); segi0486@korea.kr (E.S.C.)

**Keywords:** arginine, interleukin, interferon, heat shock protein, immune system, microbiota

## Abstract

**Simple Summary:**

Weaning stress is an economically important problem in the swine industry, and the economic loss of the growth performance reduction is even more critical if the heat stress adds to the weaning stress. The supplementation of zinc oxide (ZnO) is a promising option in reducing the adverse effects of the weaning period. However, the ban on using pharmacological doses of ZnO from 2022 in Europe will increase the postweaning issues for pig producers. Arginine is known as an anti-stress amino acid and may reduce the requirement of ZnO and adverse effects of weaning.

**Abstract:**

This study aimed to investigate the effect of the l-arginine (Arg) inclusion and different doses of ZnO on the growth performance, intestinal microbiota and integrity, and immune status of weaned pigs. A total of 180 pigs (28-day-old) were randomly allotted to six treatments with six replicate pens in each treatment and five pigs per pen. The dietary treatments were Con (1.1% Arg); P-Zn (1.1% Arg + 2500 mg Zn as ZnO/kg diet); ARG (1.6% Arg); ZnArg1 (500 mg of Zn as ZnO/kg diet + 1.6% Arg); ZnArg2 (1000 mg of Zn as ZnO/kg diet + 1.6% Arg); ZnArg3 (2500 mg of Zn as ZnO/kg diet + 1.6% Arg). The overall result showed that the inclusion of ZnArg3 significantly improved the average daily gain of pigs compared with the Con treatment. There was a reduction in feed intake in pigs fed the Con diet compared with pigs fed the ZnArg3 diet at phase 1 and overall. At phase 1, pigs fed the ZnArg3 diet and P-Zn diet showed a decreased population of *Clostridium* spp. in the ileum compared with those of the Con treatment. In addition, a lower ileal *Clostridium* spp. population was detected in pigs fed the ZnArg2 diet compared with pigs fed the Con diet. The pigs fed ZnArg1 and ZnArg3 diets showed a greater villus height of duodenum compared with the Con and P-Zn treatments. The pigs in the Con treatment showed increased mRNA expression of heat shock protein-27 in the liver compared with the P-Zn, ZnArg1, ZnArg2, and ZnArg3 treatments. When fed the basal diet, mRNA expressions of interleukin-6 were increased in the muscle compared with the ZnArg3 treatment. Dietary supplementation with ZnArg2 decreased the mRNA expressions of interferon-γ in the muscle compared with the Con treatment. Supplementation with P-Zn, ZnArg1, ZnArg2, and ZnArg3 decreased mRNA expressions of tumor necrosis factor-α (TNF-α) compared with the Con treatment. The mRNA gene expressions of interleukin-4 were decreased in the jejunum of pigs fed P-Zn, ARG, ZnArg1, ZnArg2, and ZnArg3 diets compared with pigs fed the Con diet. The jejunum gene expression of toll-like receptor-4 was upregulated in the Con and ARG treatments compared with the ZnArg1 and ZnArg3. The ZnArg1, ZnArg2, and ZnArg3 treatments showed lower mRNA expression of TNF-α compared with the Con treatment. In conclusion, there was no difference in growth performance, intestinal microbiota, gene expression of interleukins between ZnArg1 and ZnArg3 treatments. Therefore, the low level of ZnO (500 mg/kg) plus 1.6% dietary Arg may be recommended for pigs during the weaning stress.

## 1. Introduction

The health condition of piglets is highly unstable at weaning because of the stress of changing feed form, separation from the mother sow, and changes in the physical environment [1,2,3]. These stressors may cause dysbiosis due to sudden microbial changes in the intestine [1,4]. Postweaning intestinal microbiota dysbiosis is associated with some unstable situations including enteric infectious pathogens growth, leaky gut, and incomplete intestinal integrity [4]. Besides, in hot seasons, weanling pigs are under the negative influence of high temperatures, which may enhance the adverse effects of weaning stress. The low recovery rate of heat-stressed weanling piglets is mainly due to the increased oxidative damage [5]. These critical conditions impair the immune system function and intestinal mucosal development, making the piglets susceptible to oxidative stress and microbial infection.

Supplementation of pharmacological doses of ZnO (2500 mg/kg or over) in the piglet diet is a common practice among feed mills and farmers to control the adverse effects of postweaning diarrhea and increase growth performance. There is evidence that high doses of dietary ZnO controlled pathogen growth in the intestine [6]. Besides, dietary supplementation with high doses of ZnO increases the absorption rate that can enhance the antioxidant status [7]. However, feeding high doses of ZnO results in substantial quantities of Zn excretion, and causes environmental concerns [6]. The use of pharmacological doses of ZnO will be banned in June 2022 in the European Union [8], and much lower ZnO concentrations will be allowed to be used in order to reduce Zn excretion. 

It is well documented that the infectious morbidity attenuates by l-arginine (Arg) as an immune–nutrient factor [9,10]. The protective role of Arg is related to the intestinal mucosa integration and the increase in the recovery rate of the intestinal barrier [9,11]. The nutritional requirement of Arg is highly dependent on environmental and physiological conditions. Therefore, stressors such as microbial change, heat stress, and sepsis dramatically increase the Arg requirements [10,12,13]. Arginine plays a crucial role in the production of polyamine and NO, which are strong anti-stress and vasodilator factors in mammals [2]. In addition, Arg facilitates the provision of cellular signaling, intestinal recovery, and immunity factors [10,14]. Therefore, it can be hypothesized that the use of dietary Arg levels in higher doses than the recommended requirement may decrease the ZnO requirement during the stressful condition. Little information is available on the role of Arg and ZnO on oxidative damage of weanling piglets during a high ambient environment, in particular, that of immunity status, intestinal integrity, and growth performance. In this study, high dietary Arg (1.6% of the diet) and different levels of ZnO are applied to evaluate their effects on growth performance, immune status, intestinal microbiota, and morphology of pigs.

## 2. Material and Methods

The project underwent proper ethical standards and the experiments (KW-170519-1) were approved by the Institutional Animal Care and Use Committee of Kangwon National University, Chuncheon, Republic of Korea. 

### 2.1. Animals and Experimental Design

A total of 180 weaned pigs (28-day-old; Landrace × Yorkshire × Duroc; initial body weight (BW): 10.45  ±  0.03 kg) of mixed sex were randomly allotted to six treatments with 6 replicate pens in each treatment and 5 pigs per pen. The dietary treatments were Con (1.1% Arg); P-Zn (1.1% Arg + 2500 mg Zn as ZnO/kg diet); ARG (1.6% Arg); ZnArg1 (500 mg of Zn as ZnO/kg diet + 1.6% Arg); ZnArg2 (1000 mg of Zn as ZnO/kg diet + 1.6% Arg); ZnArg3 (2500 mg of Zn as ZnO/kg diet + 1.6% Arg). All the piglets were clinically healthy at the beginning of the trial. All diets (Table 1) met or exceeded the nutrient requirements according to the National Research Council (NRC) [15] containing 19% crude protein, 0.85% calcium, 0.68% phosphorus, 1.2% SID (standardized ileal digestibility) Lys, 0.39% SID Met, and 0.73% SID Met + Cys. The treatment diets were fed in a meal form in 2 phases (day 0 to 7, phase Ⅰ; and day 8 to 14, phase Ⅱ). This experiment was conducted at the facility of Kangwon National University farm and the piglets were housed in slotted and concrete floor pens with a pen size of 1.90 m × 3.0 m. All pens were equipped with a self-feeder and nipple drinker to allow ad libitum access to feed and water. Individual weanling piglet weight and feed intake from each pen were recorded at the beginning and the end of every phase to determine average daily gain (ADG), average daily feed intake (ADFI), and gain to feed ratio (G:F). All pigs were subjected to a constant room temperature of 35 °C for 10 h (07:00–17:00). The average relative humidity of the room was in the range of 43% to 59%, which indicates temperature and humidity index between 83.4 and 86.6 at the heat stress period. The rectal temperature was measured at days 1, 7, and 14 of the experiment using a digital thermometer (MSR, Measure Technology Co., Ltd.; Taipei, Taiwan). The pigs were sacrificed by electrocution followed by exsanguination. 

### 2.2. Chemical Analysis

Diet samples were analyzed in triplicate for crude protein (Method 990.03), calcium, and phosphorus (method 985.01), and ether extract (method 920.39), according to Association of Official Analytical Chemists [16]. Gross energy of diets was measured by a bomb calorimeter (Model 1261, Parr Instrument Co., Moline, IL, USA). Amino acid composition of feed samples was analyzed by High Performance Liquid Chromatography (Waters 486, Waters Corp., Milford, MA, USA) after acid hydrolysis [17]. The methionine and cysteine were determined following oxidation with performic acid [18].

### 2.3. Microbial Analyses

To study the effects of dietary treatments on small intestinal microbiota, representative piglets from each group (2 piglets per replicate; one male and one female) reflecting the average BW of the pen were selected at days 7 and 14. The digesta from the ileum was collected in sterile plastic bottles for microbial analysis. The samples collected for microbial analysis were placed on ice and immediately sent to the laboratory for analysis. The ileal digesta samples (one gram) were mixed with 9 mL peptone broth (1%) and the homogenized (Becton, Dickinson and, Franklin Lakes, NJ, USA). In the next step, serially 10-fold dilutions in the pellets were used for Viable counts of bacteria. To determine the total anaerobic bacteria (Tryptic soy agar), *Lactobacillus* spp. (using MRS agar + 0.200 g/L NaN_3_ + 0.500 g/L l-cystine hydrochloride monohydrate), *Bifidobacterium* spp. (MRS-NPNL: MRS agar + nalidixic acid, paromomycin + neomycin sulphate + lithium chloride), *Clostridium* spp. (TSC agar) and coliforms (violet red bile agar) were used. The gas pack anaerobic system (BBL, No. 260678, Difco, Detroit, MI, USA) was used for preparing anaerobic conditions. The tryptic soy agar, MRS agar, and violet red bile agar were purchased from Difco Laboratories (Detroit, MI, USA), and TSC agar (CM0589) was purchased from Oxoid (Hampshire, UK). The bacterial concentrations were transformed (log) before statistical analysis.

### 2.4. Small Intestinal Morphology 

The sacrificed pigs (two pigs per pen) were subjected to morphological tests at day 14. The intestinal morphology test was performed according to the procedure described by Hosseindoust et al. [19]. Five-centimeter cross-sectional segments were taken from the duodenum (approximately 10 cm from the pylorus), jejunum (at 50% of the total intestinal length), and ileum (at 75% of the total intestinal length). In short, for each intestinal sample, three cross-sections were prepared after staining with azure A and eosin using standard paraffin-embedding procedures. A total of 10 intact, well-oriented crypt-villus units were selected in triplicate for each intestinal cross-section. The measurement of villus height was performed from the tip of the villi to the villus–crypt junction, while the crypt depth was defined as the depth of the invagination between adjacent villi, and villus width was measured till the mid of the villus. All morphological measurements (villus height and crypt depth) were performed in 10-μm increments using an image processing and analysis system (Optimus software version 6.5, Media Cybergenetics, North Reading, MA, USA).

### 2.5. RNA Extraction and Real-Time PCR of Organ Samples

The sacrificed pigs (two pigs per pen, one male and one female) were subjected to gene expression analysis at day 14. Total RNA was isolated from the Jejunum (50 mg; from the middle jejunum segment), liver (50 mg; from the liver left lateral lobe), and muscle (50 mg; from the longissimus dorsi muscle close to the fourth rib) samples using Trizol reagent (Invitrogen, Carlsbad, CA, USA) according to the manufacturer’s instruction. Extracted RNA was quantified to 1 μg/μL and cDNA synthesis was conducted using the Improm-II Reverse transcription system (Promega, Fitchburg, MA, USA) and PCR was performed using Mx3000P real-time PCR (Stratagen, Redmond, WA, USA). The results were expressed as a relative expression by using the delta-delta method. The primers of interleukin-4 (IL-4), interleukin-6 (IL-6), interferon-γ (IFNγ), heat shock protein-27 (HSP27), toll-like receptor-4 (TLR4), and tumor necrosis factor-α (TNF-α) were presented in Table 2. In this process, β-actin was introduced to adjust the quantity of input cDNA to maintain the role in internal control [20]. A total of 20 μL reaction system included 10 μL SYBR Premix Ex Taq, 0.8 μL of forward and reverse primer (10 μM), 0.4 μL ROX Reference Dye II (50×), 2.0 μL cDNA template, and 6 μL dd H_2_O. Cycling conditions were as follows: 30 s at 95 °C, 40 cycles of denaturation step at 95 °C for 3 s, 60 °C annealing step for 34 s and a 72 °C extension step for 15 s.

### 2.6. Blood Parameters

The blood samples were collected from 2 piglets per pen on the last day of each phase. Five milliliters of ethylenediaminetetraacetic acid (EDTA) treated (Becton Dickinson, Franklin Lakes, NJ, USA) and not-treated blood samples were collected from the jugular vein and stored on ice for immediate hematological analysis. The EDTA-treated blood was diluted by Natt-Herrick solution and mixed for 15 min for white blood cells (WBC), red blood cells (RBC), lymphocytes, and monocytes analyses using Hemavet Multispecies Hematology Systems (Scientific Inc., Oxford, CT, USA). The rest of the blood samples were centrifuged at 1500 rpm for 20 min in a centrifuge, and plasma was separated and used for cortisol analysis. Cortisol was analyzed using an ELISA kit (ADI-900-70; Enzo Life Sciences, Farmingdale, NY, USA).

### 2.7. Statistical Analysis

The data were analyzed as a completely randomized design using the GLM procedure of SAS (version 9.4, 1996, SAS Inst. Inc., Cary, NC, USA). The initial body weight was used as a covariate for growth performance but was removed from the model when not significant. Each piglet was an experimental unit for growth performance, feed intake, microbial test, intestinal morphology, blood parameters, and gene expression analyses. The Tukey means comparison test was applied for treatment means separation by at *p* < 0.05 statistical level. Probability level of less than 0.1 was considered a tendency.

## 3. Results

### 3.1. Rectal Temperature and Growth Performance 

The average rectal temperature measured at days 1, 7, and 14 of the trial were 38.5 ± 0.82 °C, 38.1 ± 0.76 °C, and 38.1 ± 0.84 °C, respectively. The results of growth performance are shown in Table 3. There was no difference in the ADG of pigs in the first and second phases. The overall result showed that the inclusion of ZnArg3 significantly improved (*p* < 0.05) the ADG compared with the Con treatment. There was a reduction in ADFI in pigs in the Con treatment compared with the pigs fed the ZnArg3 diet in phase 1 and overall. The gain to feed ratio was not affected by the treatments.

### 3.2. Microflora Composition

The microbial population in the ileum is shown in Table 4. There was no difference in the population of total anaerobic bacteria, *Bifidobacterium* spp., *Lactobacillus* spp., and Coliforms among the treatments. At phase 1, pigs in ZnArg3 and P-Zn treatments showed a decreased population of *Clostridium* spp. in the ileum compared with those of the Con treatment (*p* < 0.01). In addition, a lower ileal *Clostridium* spp. population was detected in pigs fed ZnArg2 diet compared with pigs fed Con diet. At phase 2, the colonization of *Clostridium* spp. was higher in the Con and ARG treatments compared with the ZnArg3 treatment.

### 3.3. Intestinal Morphology

As shown in Table 5, pigs fed ZnArg1 and ZnArg3 diets had greater villus height in the duodenum compared with pigs fed Con and P-Zn diets. Moreover, there was a tendency of higher villus height in the jejunum of pigs fed the ZnArg3 diet compared with pigs fed the Con diet. There was no difference in villus height in the jejunum and ileum. In addition, the crypt depth and villus height to crypt depth ratio were not affected by the dietary treatments. 

### 3.4. Blood Parameters

The results of the blood parameters are presented in Table 6. The concentration of WBC, RBC, and cortisol was not affected by the treatments. The number of lymphocytes was not affected at day 7, however, there was a lower number of lymphocytes in ZnArg1, ZnArg2, and ZnArg3 treatments compared with Con treatment at day 14. There was no difference among the treatments for the number of monocytes, however, eosinophil number was higher (*p* = 0.021) in Con and ZnArg1 treatments compared with ZnArg2 and ZnArg3 treatments. There was a tendency to lower blood cortisol in pigs fed ZnArg2 diet compared with pigs fed Con diet.

### 3.5. Gene Expression in the Organs

**Figure 1:**
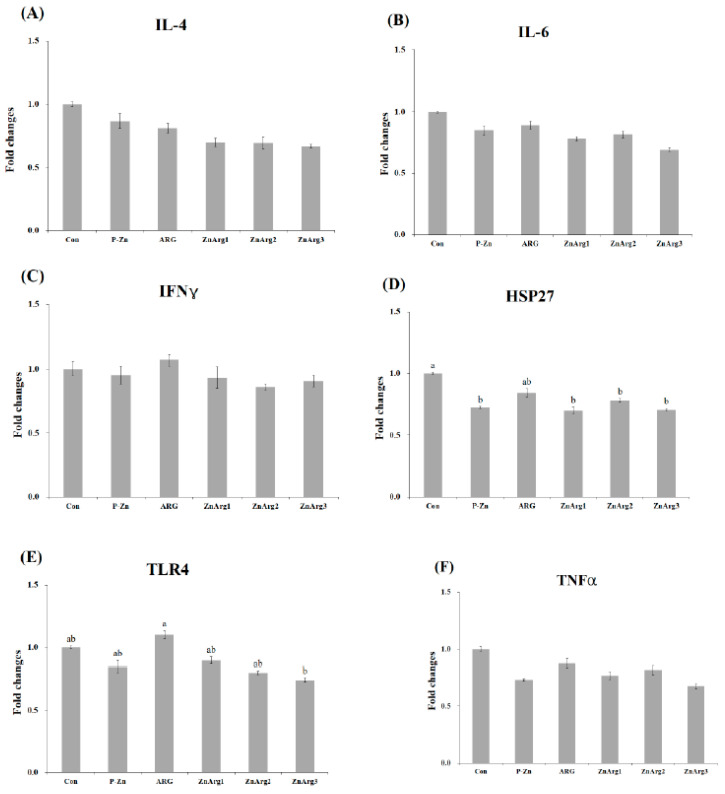
Relative expression of, (**A**) interleukin-4 (IL-4), (**B**) interleukin-6 (IL-6), (**C**) interferon-γ (IFNγ), (**D**) heat shock protein-27 (HSP27), (**E**) toll like receptor-4 (TLR4), and (**F**) tumor necrosis factor-α (TNF-α) in the liver of weanling piglets. Error bars represent standard error of means. Bars with different letters (a~b) differ significantly across all 6 treatment groups (*p* < 0.05). Con: 1.1% Arg; P-ZnO: 1.1% Arg + 2500 mg Zn/kg diet; ARG: 1.6% Arg; ZnArg1: 1.6% Arg + 500 mg Zn/kg diet; ZnArg2: 1.6% Arg + 1000 mg Zn/kg diet; ZnArg3: 1.6% Arg + 2500 mg Zn/kg diet.

**Figure 2:**
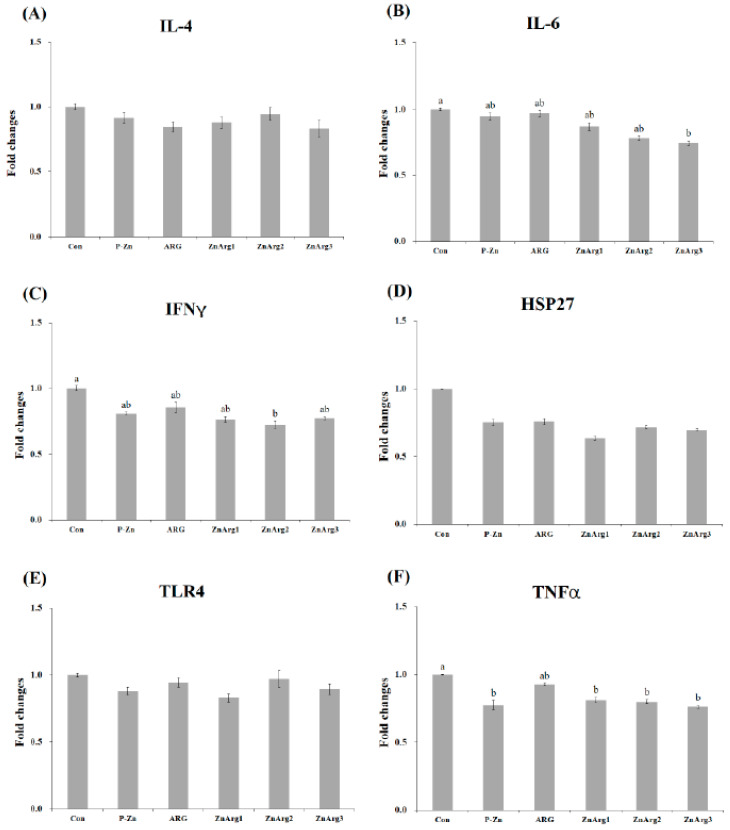
Relative expression of, (**A**) interleukin-4 (IL-4), (**B**) interleukin-6 (IL-6), (**C**) interferon-γ (IFNγ), (**D**) heat shock protein-27 (HSP27), (**E**) toll like receptor-4 (TLR4), and (**F**) tumor necrosis factor-α (TNF-α) in the liver of weanling piglets. Error bars represent standard error of means. Bars with different letters (a~b) differ significantly across all 6 treatment groups (*p* < 0.05). Con: 1.1% Arg; P-ZnO: 1.1% Arg + 2500 mg Zn/kg diet; ARG: 1.6% Arg; ZnArg1: 1.6% Arg + 500 mg Zn/kg diet; ZnArg2: 1.6% Arg + 1000 mg Zn/kg diet; ZnArg3: 1.6% Arg + 2500 mg Zn/kg diet.

**Figure 3:**
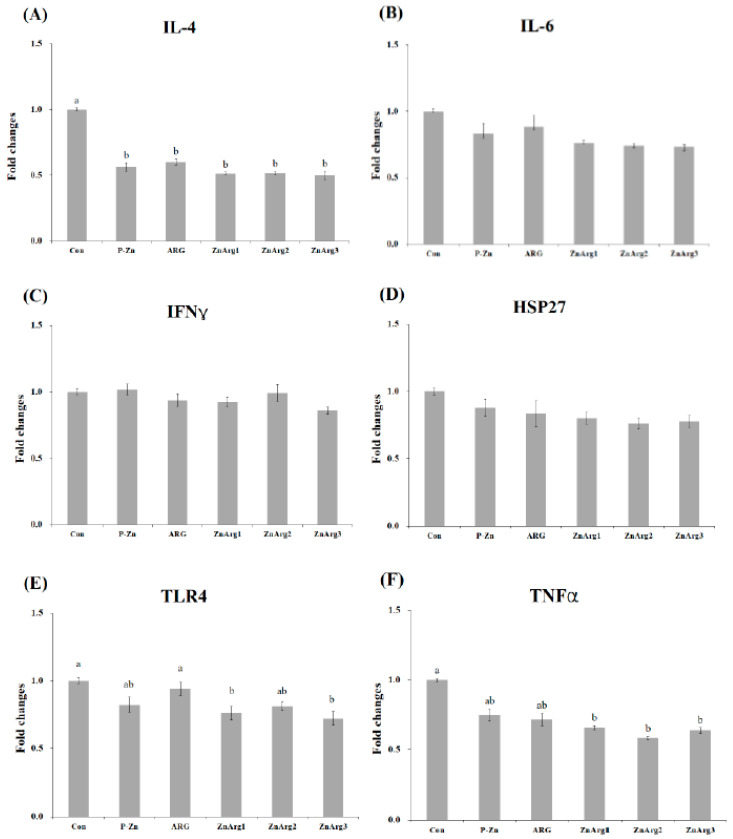
Relative expression of, (**A**) interleukin-4 (IL-4), (**B**) interleukin-6 (IL-6), (**C**) interferon-γ (IFNγ), (**D**) heat shock protein-27 (HSP27), (**E**) toll like receptor-4 (TLR4), and (**F**) tumor necrosis factor-α (TNF-α) in the liver of weanling piglets. Error bars represent standard error of means. Bars with different letters (a~d) differ significantly across all 6 treatment groups (*p* < 0.05). Con: 1.1% Arg; P-ZnO: 1.1% Arg + 2500 mg Zn/kg diet; ARG: 1.6% Arg; ZnArg1: 1.6% Arg + 500 mg Zn/kg diet; ZnArg2: 1.6% Arg + 1000 mg Zn/kg diet; ZnArg3: 1.6% Arg + 2500 mg Zn/kg diet.

The pigs in the Con treatment showed higher mRNA expression of *HSP27* in the liver compared with the P-Zn, ZnArg1, ZnArg2, and ZnArg3 treatments (*p* < 0.05, Figure 1). Greater gene expression of TLR4 was observed in pigs fed ARG compared with pigs fed ZnArg3. There were no differences between the treatments in the gene expression of IL-4, IL-6, IFNγ, and TNF-α in the liver. When fed the Con diet, pigs had enhanced mRNA expressions of IL-6 in the muscle compared with pigs fed ZnArg3 (*p* < 0.05, Figure 2). Dietary supplementation with 1.6% Arg and 1000 mg/kg Zn as ZnO decreased the mRNA expressions of IFNγ in the muscle compared with Con treatment (*p* < 0.05). TNF-α expression was decreased in pigs fed P-Zn, ZnArg1, ZnArg2, and ZnArg3 compared with pigs fed Con (*p* < 0.05). There were no differences among the treatments in the gene expression of IL-4, HSP27, and TLR4. The mRNA gene expressions of IL-4 were decreased in the jejunum of pigs fed P-Zn, ARG, ZnArg1, ZnArg2, and ZnArg3 compared with pigs fed Con (Figure 3; *p* < 0.05). The ZnArg1, ZnArg2, and ZnArg3 treatments showed lower mRNA expression of TNF-α compared with the Con treatment. The gene expression of TLR4 in the jejunum was upregulated in the Con and ARG treatments compared with the ZnArg1 and ZnArg3 treatments. The treatments did not significantly impact the mRNA expression of IL-6, IFNγ, and HSP27 in the jejunum.

## 4. Discussion

The supplementation of pharmacological doses of ZnO is routinely considered in weaned pigs’ diets to alleviate diarrhea incidence and reduce growth depression during the weaning period [6,21]. In our study, the response to ADG and ADFI in pigs fed the ZnArg3 diet was increased relative to the pigs fed the Con diet, however, there were no differences between the ARG and ZnArg3 treatments. Compromising the growth performance in pigs fed the Con diet could be resulting from the insufficient nutrients intake, as shown by the significantly reduced ADFI in the Con treatment. The finding that supplementing 2500 mg/kg of Zn as ZnO showed no growth benefit compared with pigs fed 500 or 1000 mg/kg ZnO was in contrast to a previous study, which reported a significant difference between 2500 mg/kg ZnO and levels less than 1000 mg/kg [22]. It is believed that the lower doses of ZnO (below 1000 mg/kg) are not effective in the performance of weaned pigs [6,23]. The literature shows that the pharmacological dose of ZnO (2500 mg/kg or above) increases ADG and ADFI due to controlling the population of *Escherichia coli*, *clostridium*, and incidence of diarrhea [9,24]. Pigs fed the ZnArg3 diet were less under stress due to the lower gene expression of HSP27, which can be responsible for higher ADFI. In addition, our results showed that the ADG and ADFI of pigs in ZnArg1 treatments were comparable with pigs in ZnArg3 treatment. It may be postulated that the positive anti-stress effects of dietary Arg may be responsible for insignificant differences between ZnArg3 and ZnArg1 treatments. Anti-stress effects of Arg may have a complementary role for the pharmacological dose of ZnO in case of decreasing stress, as Arg activates the production of nitric oxide and peroxynitrite [25]. However, our results rejected the hypothesis of the direct effects of Arg in eliminating pathogens [26] due to the lack of difference in the number of coliforms and clostridia in the ARG treatment, and only the pharmacological dose of ZnO (2500 mg/kg) showed a definite effect in controlling *Clostridium* spp. The decreased villus height in pigs fed the Con diet may be another reason for the lower growth performance, which may be related to the anti-pathogenic properties of ZnO.

During weaning stress, the manipulation of microbial population in order to control opportunistic pathogens becomes a priority. Moreover, it is widely accepted that intestinal mucosal integrity is generally associated with gut microbiota. This interaction might be of importance when animals have to struggle with the change of diet form and facing a severe change in the microbial biomass and composition in the intestine. Although in the current study the absorption of Zn and the concentration of ZnO in the digesta were not studied, the microbiota composition in different phases was evaluated. A lower number of *Clostridium* spp. was detected in pigs fed the ZnArg3 diet compared with pigs fed the Con diet, however, the lower ZnO supplementations (ZnArg1 and ZnArg2) did not show a significant effect on *Clostridium* spp. colonization. Hosseindoust et al. [24] and Liu et al. [7] reported that the antimicrobial activity of ZnO against coliforms makes it an ideal candidate to control the microbiota, however, they did not report any change in the number of *Clostridium* spp. In another study, the population of coliforms and *Clostridium* spp. was linearly decreased in the ileum and colon of weaned pigs when the dose of ZnO increased from 500 mg/kg to 2500 mg/kg [6]. It is reasonable to speculate that influences on the intestinal microbiota might not be modified by ZnO with lower doses than the pharmacological one. Additionally, the supplementation with 1.6% Arg did not modify the microbial population. This result disagrees with the report of Ren et al. [27], who supplemented a high dose of Arg containing 0.93% l-arginine gram into the diet of mice to manipulate the microbial change in favor of beneficial bacteria including *Lactobacillus* spp. It is crucial to investigate which ratio of Arg and ZnO will influence the weaned pig’s microbiota and immune status.

In the present study, a severe decrease in villus height in the duodenum occurred in pigs fed the Con diet compared with pigs fed ZnArg1 and ZnArg3. Intriguingly, the villus height in the jejunum and ileum remained unchanged. Kim et al. [6] reported a greater villus height in the duodenum and ileum of weaned pigs by the supplementation of a pharmacological dose of ZnO into the diet. The reduced villus crypt structure alteration including villus height and crypt depth, crypt hyperplasia, and generally intestinal structural injury occurs mostly during the weaning [28]. The villus height was not improved in pigs fed ARG diet. The role of different doses of Arg on intestinal morphology should be further studied since the weaning time is the most critical period.

The mRNA expression levels of HSP27 in the livers were remarkably decreased in pigs fed P-Zn, ZnArg1, ZnArg2, and ZnArg3 diets compared with the Con treatment. The positive correlation between HSP expression and stress was previously reported in other studies [2,4,13]. The low gene expression of HSP27 in the liver and a tendency for lower blood cortisol in the ZnArg2 and ZnArg3 treatments may indicate a lower stress level compared with the Con treatment. Arg is reported to be an anti-stress or immunomodulatory factor by the activation of ornithine decarboxylase and generating polyamines [10]. In addition, to our knowledge, there is no report related to the influence of dietary Arg on the TLR gene expression in weanling pigs during heat stress. In this study, a high level (1.6% of diet) of dietary Arg increased the gene expression of TLR4 in the jejunum and liver. These findings may be explained by the result of a microbial test wherein dietary Arg showed significantly higher *Clostridium* spp. population compared with ZnO-supplemented treatments in the small intestine. A lower number of *Clostridium* spp. was detected in pigs fed the ZnArg3 diet compared with pigs fed the Con diet, which might have caused a decrease in TLR4 expression in the jejunum. The pathogenic microbes are recognized by the innate immune system through the stimulation of TLRs. The TLR4 is a type of pattern recognition receptors in epithelial cells that have been identified as the main pathway on the innate immune system for recognition of lipopolysaccharide (LPS), a component of the bacterial cell wall [29]. The over-expression of the TLR signaling pathway may be responsible for the stress-induced suppression of the immune system. Interestingly, our result supports the hypothesis that cytokines production increases through TLR4 signaling among ZnO-supplemented treatments by increasing the gene expression of IL-4 in the jejunum and IL-4 and TNF-α in the liver. Dietary ZnO beneficially affected the bacterial communities and mRNA expression of inflammatory cytokines and TLR4.

A large body of evidence has shown the upregulation of pro-inflammatory cytokine gene expression within a short time of weaning [28,30,31]. In the present study, the expression of TNF-α was decreased in the jejunum of pigs fed P-Zn, ZnArg1, ZnArg2, and ZnArg3 diets. Intestinal inflammation occurs in pigs after weaning [23]. The high inflammation induces an elevated specific secretion of cytokines from intestinal lymphocytes [32]. The current study showed a lower expression of IL-4 in the jejunum and a lower count of lymphocytes in pigs fed ZnArg1, ZnArg2, and ZnArg3 diets. IL-4 is known as a B lymphocyte growth promoter and can linearly modulate the production and development of B and T lymphocytes [33]. In contrast, Han et al. [10] reported that the content of IL-2 and IFNγ in the serum was increased in cyclophosphamide-challenged weaned piglets. The over-production of TNF-α and IL-6 compromise the intestinal epithelial permeability, resulting in increased pathologic opening in the tight junction [22]. The extra-cellular pathogens, such as intestinal microbes, secrete IL-4, IL-10, and IL-13 to activate the immune system and produce strong antibody-mediated responses [34]. In agreement, it has long been known that the IL-6 concentration increases in stressful conditions and during acute inflammation [35]. The increased gene expressions of TNF-α, IL-6, and IFNγ were noticed in salmonella-challenged pigs [36]. It has been reported that lactic acid bacteria downregulate the gene expression of inflammatory cytokines [37]. In addition, the higher number of eosinophils in pigs fed the Con diet may be responsible for higher gene expression of cytokines. It has been reported that the eosinophils increase the gene expression of IL-1, IL-4, IL-6, and TNF-α [38]. As the increase in pro-inflammatory cytokines adversely affects the development of intestinal epithelium, controlling the concentration of intestinal inflammatory cytokines and intestinal integrity is crucial to alleviate intestinal disorders at the weaning time. 

## 5. Conclusions

The growth performance, intestinal microbiota, microbial composition, and immune status of weanling pigs were improved in pigs fed P-Zn, ZnArg1, ZnArg2, and ZnArg3 diets but not in pigs fed the ARG diet. However, the heat-challenged weaned piglets fed diets supplemented with 1.6% Arg and low ZnO (500 mg/kg) content expressed similar growth performance, intestinal microbiota, intestinal integrity, as those fed diets supplemented with 1.6% Arg and high ZnO (2500 mg/kg) content. Therefore, it can be postulated that the dietary Arg alone cannot be effective during the weaning stress but can be practically applicable to dietary ZnO supplementation. Further studies are required to determine the optimum combination of Arg and ZnO.

## Figures and Tables

**Table 1:** Formula and chemical composition of basal diets (as-fed basis).

Treatments	Con	P-Zn	ARG	ZnArg1	ZnArg2	ZnArg3
Zinc Oxide (mg/kg Diet)	0	2500	0	500	1000	2500
Arginine (%)	1.1	1.1	1.6	1.6	1.6	1.6
Corn	49.79	49.48	49.29	49.23	49.17	48.98
SBM dehulled	17.20	17.20	17.20	17.20	17.20	17.20
Fish Chile	3.00	3.00	3.00	3.00	3.00	3.00
Whey (Korea)	10.00	10.00	10.00	10.00	10.00	10.00
Lactose	6.00	6.00	6.00	6.00	6.00	6.00
SDPP	4.00	4.00	4.00	4.00	4.00	4.00
Sugar	4.00	4.00	4.00	4.00	4.00	4.00
Soy Oil	3.00	3.00	3.00	3.00	3.00	3.00
l-Lysine 78%	0.09	0.09	0.09	0.09	0.09	0.09
dl-Methionine 100%	0.06	0.06	0.06	0.06	0.06	0.06
l-Tryptophan 10%	0.18	0.18	0.18	0.18	0.18	0.18
Arg (100%)	-	-	0.50	0.50	0.50	0.50
Limestone	1.1	1.1	1.1	1.1	1.1	1.1
MCP	0.82	0.82	0.82	0.82	0.82	0.82
Salt	0.43	0.43	0.43	0.43	0.43	0.43
ZnO 78%	0	0.31	0	0.06	0.12	0.31
Vitamin premix ^1^	0.11	0.11	0.11	0.11	0.11	0.11
Mineral premix ^2^	0.22	0.22	0.22	0.22	0.22	0.22
Total	100	100	100	100	100	100
Analyzed composition (%)						
Crude protein	18.95	18.82	18.86	18.88	18.92	18.91
Crude fat	1.32	1.26	1.29	1.24	1.31	1.30
Ca	0.85	0.87	0.87	0.86	0.85	0.85
P	0.70	0.66	0.69	0.68	0.69	0.67
Arg	1.16	1.13	1.64	1.65	1.63	1.65
Lys	1.48	1.52	1.48	1.49	1.45	1.47
Met	0.43	0.42	0.401	0.44	0.42	0.44
Thr	0.90	0.93	0.92	0.92	0.92	0.92
Zn	0.085	2.64	0.079	0.51	1.03	2.58

Abbreviations: Con: 1.1% Arg; P-ZnO: 1.1% Arg + 2500 mg Zn/kg diet; ARG: 1.6% Arg; ZnArg1: 1.6% Arg + 500 mg Zn/kg diet; ZnArg2: 1.6% Arg + 1000 mg Zn/kg diet; ZnArg3: 1.6% Arg + 2500 mg Zn/kg diet; SBM: soybean meal; SDPP: spray-dried porcine plasma; MCP: mono calcium phosphate; ^1^ supplied per kilogram of diet: 16,000 IU vitamin A, 3000 IU vitamin D_3_, 40 IU vitamin E, 5.0 mg vitamin K_3_, 5.0 mg vitamin B_1_, 20 mg vitamin B_2_, 4 mg vitamin B_6_, 0.08 mg vitamin B_12_, 40 mg pantothenic acid, 75 mg niacin, 0.15 mg biotin, 0.65 mg folic acid. ^2^ Supplied per kilogram of diet: 45 mg Fe, 0.25 mg Co, 50 mg Cu, 15 mg Mn, 25 mg Zn, 0.35 mg I, 0.13 mg Se.

**Table 2:** Primers.

Gene	Primer Sequence (5⟶3)
*β-actin_F*	CAACACAGTGCTGTCTGGTGGTA
*β-actin_R*	ATCGTACTCCTGCTTGCTGATCC
*IL4_F*	TGTGCCCACGCTGTGCTTACA
*IL4_R*	CTTGTGGCAGTGCTGGCTCTCC
*IL6_F*	AGAAATCCCTCCTCGCCAAT
*IL6_R*	AAATAGCGAACGGCCCTCA
*IFNγ_F*	CTGAAGAACTGGACAGAGAG
*IFNγ_R*	CACCAGCTTCTGTAAGATGC
*HSP27_F*	GGAGATCACCGGCAAACACG
*HSP27_R*	CCTCCACTGTCAGCATCCCA
*TLR4_F*	GTCTCTCCTTCCTTACCTGCTGTTC
*TLR4_R*	AGGAGGAGAAAGACAGGGTAGGTG
*TNFα-F*	GGATCATCTTCTCGAACCCCGAGTGACAAG
*TNFα-R*	GTTCAGGACGCAGACAATGTTCT

Abbreviations: IL, interleukin; IFN, interferon; HSP, heat shock protein; TLR, toll-like receptor; TNF, tumor necrosis factor.

**Table 3:** Effect of dietary zinc and arginine on body weight (BW) average daily gain (ADG), average daily feed intake (ADFI), feed efficiency (gain:feed) of weanling piglets exposed to high temperature.

Treatments	Con	P-Zn	ARG	ZnArg1	ZnArg2	ZnArg3	SEM	*p*-Value
Zinc Oxide (mg/kg Diet)	0	2500	0	500	1000	2500		
Arginine (%)	1.1	1.1	1.6	1.6	1.6	1.6		
Initial BW, kg	10.42	10.46	10.44	10.48	10.49	10.42	0.111	0.995
7 days BW, kg	12.37	12.53	12.57	12.67	12.66	12.72	0.121	0.191
14 days BW, kg	14.45	14.71	14.78	15.03	15	15.16	0.247	0.396
ADG								
0 to 7 d	278	296	305	313	310	329	17.11	0.429
8 to 14 d	297	311	316	338	335	348	27.98	0.8
0 to 14 d	288 ^b^	304 ^a,b^	310 ^a,b^	325 ^a,b^	322 ^a,b^	340 ^a^	10.94	0.034
ADFI								
0 to 7 d	458 ^b^	476 ^a,b^	482 ^a,b^	507 ^a,b^	489 ^a,b^	533 ^a^	16.36	0.048
8 to 14 d	514	525	512	532	539	560	23.67	0.718
0 to 14 d	486 ^b^	501 ^a,b^	497 ^a,b^	520 ^a,b^	514 ^a,b^	547 ^a^	13.44	0.05
Gain:feed ratio								
0 to 7 d	0.607	0.623	0.631	0.615	0.634	0.617	0.030	0.985
8 to 14 d	0.578	0.591	0.614	0.627	0.619	0.62	0.038	0.934
0 to 14 d	0.592	0.606	0.624	0.625	0.626	0.623	0.050	0.78

^a,b^ Means in the same line with different superscripts differ significantly (*p* < 0.05). Con: 1.1% Arg; P-ZnO: 1.1% Arg + 2500 mg Zn/kg diet; ARG: 1.6% Arg; ZnArg1: 1.6% Arg + 500 mg Zn/kg diet; ZnArg2: 1.6% Arg + 1000 mg Zn/kg diet; ZnArg3: 1.6% Arg + 2500 mg Zn/kg diet; SEM: standard error of mean; BW: body weight; ADG: average daily gain; ADFI: average daily feed intake.

**Table 4:** Effect of dietary zinc and arginine on intestinal microbiota of weanling piglets fed diets supplemented with or without zinc and arginine exposed to high temperature.

Treatments	Con	P-Zn	ARG	ZnArg1	ZnArg2	ZnArg3	SEM	*p*-Value
Zinc Oxide (mg/kg Diet)	0	2500	0	500	1000	2500		
Arginine (%)	1.1	1.1	1.6	1.6	1.6	1.6		
day 7								
Total anaerobic bacteria	8.84	8.89	8.86	8.73	8.82	8.69	0.117	0.838
*Bifidobacterium* spp.	7.66	7.72	7.71	7.63	7.74	7.8	0.154	0.975
*Lactobacillus* spp.	8.63	8.66	8.64	8.52	8.6	8.62	0.070	0.725
*Clostridium* spp.	8.61 ^a^	8.23 ^b^	8.51 ^a,b^	8.38 ^a,b^	8.34 ^a,b^	8.19 ^b^	0.062	0.001
Coliforms	8.39	8.23	8.27	8.21	8.22	8.21	0.048	0.115
day 14								
Total anaerobic bacteria	8.61	8.64	8.65	8.55	8.61	8.77	0.102	0.731
*Bifidobacterium* spp.	7.47	7.6	7.52	7.49	7.42	7.66	0.134	0.835
*Lactobacillus* spp.	8.38	8.39	8.33	8.28	8.29	8.45	0.081	0.588
*Clostridium* spp.	8.54 ^a^	8.24 ^a,b^	8.41 ^a^	8.27 ^a,b^	8.34 ^a,b^	8.03 ^b^	0.073	0.003
Coliforms	8.32	8.25	8.33	8.24	8.29	8.2	0.039	0.128

^a,b^ Means in the same line with different superscripts differ significantly (*p* < 0.05). Con: 1.1% Arg; P-ZnO: 1.1% Arg + 2500 mg Zn/kg diet; ARG: 1.6% Arg; ZnArg1: 1.6% Arg + 500 mg Zn/kg diet; ZnArg2: 1.6% Arg + 1000 mg Zn/kg diet; ZnArg3: 1.6% Arg + 2500 mg Zn/kg diet; SEM: standard error of mean.

**Table 5:** Effect of dietary zinc and arginine on intestinal morphology of weanling piglets exposed to high temperature.

Treatments	Con	P-Zn	ARG	ZnArg1	ZnArg2	ZnArg3	SEM	*p*-Value
Zinc Oxide (mg/kg Diet)	0	2500	0	500	1000	2500		
Arginine (%)	1.1	1.1	1.6	1.6	1.6	1.6		
Villus height (VH, μm)								
Duodenum	528 ^b^	550 ^a,b^	567 ^a,b^	606 ^a^	587 ^a,b^	676 ^a^	27.94	0.014
Jejunum	459	508	495	532	501	583	28.74	0.095
Ileum	391	408	432	438	433	453	15.33	0.101
Crypt depth (CD, μm)								
Duodenum	341	333	315	328	334	346	15.22	0.768
Jejunum	243	265	283	280	260	271	18.37	0.665
Ileum	235	254	253	253	256	253	11.71	0.576
VH/CD								
Duodenum	1.56	1.67	1.82	1.84	1.79	1.97	0.111	0.21
Jejunum	1.91	1.92	1.84	1.93	1.97	2.17	0.142	0.658
Ileum	1.69	1.62	1.67	1.77	1.79	1.8	0.109	0.804

^a,b^ Means in the same line with different superscripts differ significantly (*p* < 0.05). Con: 1.1% Arg; P-ZnO: 1.1% Arg + 2500 mg Zn/kg diet; ARG: 1.6% Arg; ZnArg1: 1.6% Arg + 500 mg Zn/kg diet; ZnArg2: 1.6% Arg + 1000 mg Zn/kg diet; ZnArg3: 1.6% Arg + 2500 mg Zn/kg diet; SEM: standard error of mean.

**Table 6:** Effect of dietary zinc and arginine on blood characteristics of weanling piglets exposed to high temperature.

Treatments	Con	P-Zn	ARG	ZnArg1	ZnArg2	ZnArg3	SEM	*p*-Value
Zinc Oxide (mg/kg Diet)	0	2500	0	500	1000	2500		
Arginine (%)	1.1	1.1	1.6	1.6	1.6	1.6		
WBC, 10^3^/uL								
Day 7	9.40	9.78	9.82	10.09	10.23	10.12	0.321	0.435
Day 14	13.74	16.11	15.68	16.53	16.15	16.09	1.431	0.777
RBC, 10^6^/uL								
Day 7	7.46	7.72	7.7	7.92	7.6	7.85	0.296	0.906
Day 14	7.93	8.17	7.11	7.51	7.06	7.72	0.330	0.132
Lymphocytes, %								
Day 7	74.13	71.56	72.54	70.97	70.72	70.66	1.070	0.192
Day 14	77.94 ^a^	71.54 ^a,b^	71.03 ^a,b^	68.65 ^b^	69.36 ^b^	69.45 ^b^	1.871	0.016
Monocytes, %								
Day 7	3.95	3.3	3.18	3.12	3.07	3.03	0.322	0.353
Day 14	4.6	4.51	4.43	3.85	3.28	4.23	0.348	0.101
Eosinophil, %								
Day 7	2.1	2.32	2.13	1.98	2.07	2.13	0.150	0.766
Day 14	3.57 ^a^	2.78 ^a,b^	2.95 ^a,b^	3.40 ^a^	2.52 ^b^	2.63 ^b^	0.243	0.021
Cortisol, ug/dL								
Day 7	5.17	4.8	4.58	4.12	4.37	4.43	0.331	0.3
Day 14	5.39	4.53	4.88	4.62	4.11	4.2	0.240	0.086

^a,b^ Means in the same line with different superscripts differ significantly (*p* < 0.05). Con: 1.1% Arg; P-ZnO: 1.1% Arg + 2500 mg Zn/kg diet; ARG: 1.6% Arg; ZnArg1: 1.6% Arg + 500 mg Zn/kg diet; ZnArg2: 1.6% Arg + 1000 mg Zn/kg diet; ZnArg3: 1.6% Arg + 2500 mg Zn/kg diet; SEM: standard error of mean; RBC: red blood cell; WBC: white blood cells.

## Abbreviations

ARG	arginine supplementation
TNF	tumor necrosis factor
IL	interleukin
INF	interferon
HSP	heat shock protein
TLR	toll-like receptor
LPS	lipopolysaccharide
ADG	average daily gain
ADFI	average daily feed intake
GF	gain to feed ratio
WBC	white blood cells
RBC	red blood cells
P-Zn	2500 mg/kg of Zn as ZnO
ZnArg1	500 mg Zn as ZnO/kg diet + 1.6% Arg
ZnArg2	1000 mg Zn as ZnO/kg diet + 1.6% Arg
ZnArg3	2500 mg Zn as ZnO/kg diet + 1.6% Arg
NO	nitrogen oxide
